# Blastic Plasmacytoid Dendritic Cell Neoplasm: A Case Report

**DOI:** 10.30699/ijp.2025.2025501.3279

**Published:** 2025-08-15

**Authors:** Farzaneh Nayeri, Pardis Nematolahi, Behnoosh Mohamadi

**Affiliations:** 1 *Department of Pathology, Resident of Pathology, Isfahan University of Medical Sciences, Isfahan, Iran *; 2 *Department of Pathology, Associate Professor, Isfahan University of Medical Sciences, Isfahan, Iran *; 3 *Department of Pathology, Assistant Professor, Isfahan University of Medical Sciences, Isfahan, Iran*

**Keywords:** Acute leukemia, Blastic plasmacytoid dendritic cell neoplasm, Chemotherapy, Stem cell transplantation

## Abstract

A rare condition, blastic plasmacytoid dendritic cell neoplasm, is classified as acute myeloid leukemia–related precursor neoplasms according to the World Health Organization’s 2022 classification. Previously thought to originate from natural killer cells, T cells, or monocytes, it is now believed to arise from plasmacytoid dendritic cells. The cause of this condition is not well understood, but it is often associated with the deletion of tumor suppressor genes such as RB1, CDKN1B, CDKN2A, and TP53.

The disease is aggressive and typically presents with initial cutaneous lesions that can progress to bone marrow involvement and leukemic dissemination. Flow cytometry/ immunohistochemistry can detect enhanced CD56, CD4, and CD123 expression. The differential diagnoses include myeloid sarcoma/acute myeloid leukemia, T-cell lymphoblastic leukemia/lymphoma, NK-cell lymphoma/leukemia, and certain mature T-cell lymphomas/leukemias. Although initial chemotherapy may elicit a patient response, relapse is common. Survival may be improved by stem cell transplantation.

This case report details the medical history of a 64-year-old woman who presented with a skin mass that exhibited slow growth over 6 months. The mass was firm on palpation. Extensive testing, including a bone marrow (BM) smear and biopsy, revealed numerous abnormal or blastic cells. Furthermore, flow cytometric analysis of the BM confirmed the presence of plasmacytoid dendritic cell–neoplastic precursor cells exhibiting CD4+ and CD56+ characteristics.

## Introduction

In 1994, a medical condition called blastic plasmacytoid dendritic cell neoplasm (BPDCN) was first recognized as not very common (1). The lack of understanding regarding the histogenesis of BPDCN has led to various classifications of the disease, such as a tumor, blastic natural killer (NK) leukemia/lymphoma, agranular CD4+ NK cell leukemia, or agranular CD4+ CD56+ hematodermic neoplasm (2,3). The 2022 World Health Organization classification assigns BPDCN to the category of precursor neoplasms associated with acute myeloid leukemia (4). The main manifestation of the illness is characterized by numerous cutaneous lesions at diagnosis, which can then gradually extend to involve the peripheral blood, bone marrow, and lymph nodes. The clinical behavior of BPDCN is aggressive and often results in poor survival rates.

## Case Presentation

**Fig. 1:**
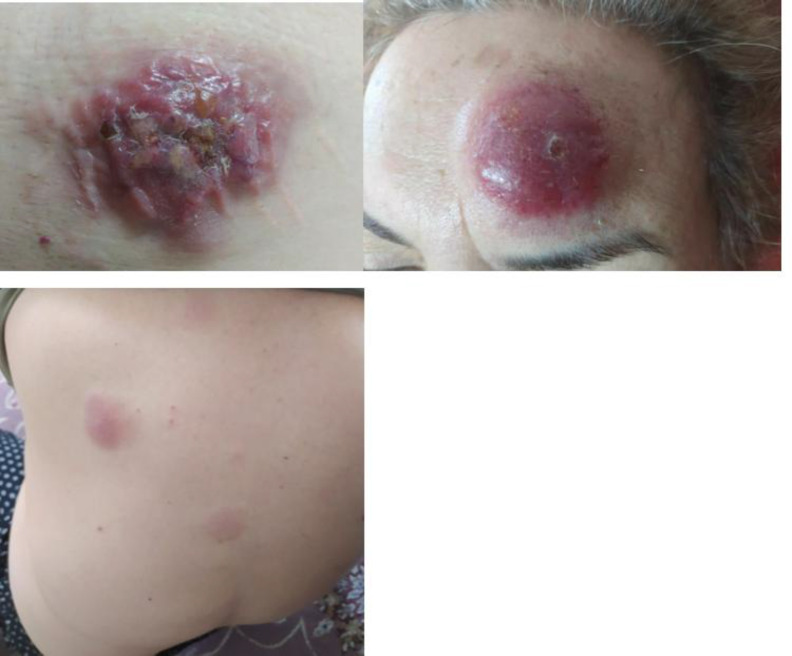
Macroscopic view of skin lesions

A 64-year-old woman presented with a 6-month history of several slow-growing, indurated, pruritic, and ulcerated skin masses on the face and the anteromedial aspect of the trunk. These lesions were tender and mobile. The largest mass measured 10 × 5 cm, with ulceration of the surface skin and purulent drainage, likely due to wound superinfection (Fig 1). The patient had no significant medical history, although her family history included hemophilia and breast cancer. A routine blood count was within normal limits (WBC 4300/µL; hemoglobin 12.9 g/dL; platelets 229,000/µL). On physical examination, there was no organomegaly or lymphadenopathy. Her MDCT was normal, but an abdominal CT scan showed three lymphadenopathies in the right inguinal canal.

**Fig. 2a:**
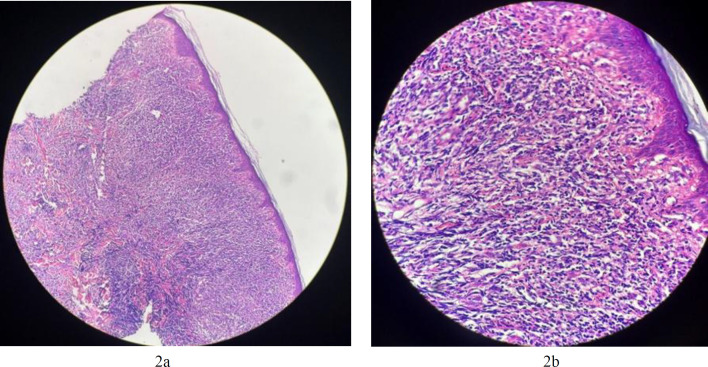
Microscopic view of skin lesion H&E × 4. A monotonous population of intermediate-sized cells infiltrated the dermis and subcutis; **2b.** Microscopic view of skin lesion H&E ×10

A biopsy of the skin lesion revealed a monotonous population of intermediate-sized cells infiltrating the dermis and subcutis, with irregular nuclear contours and dispersed chromatin in the background of mixed small B and T cells. The overlying epidermis was not involved (Figs 2a and 2b).

**Fig.3a:**
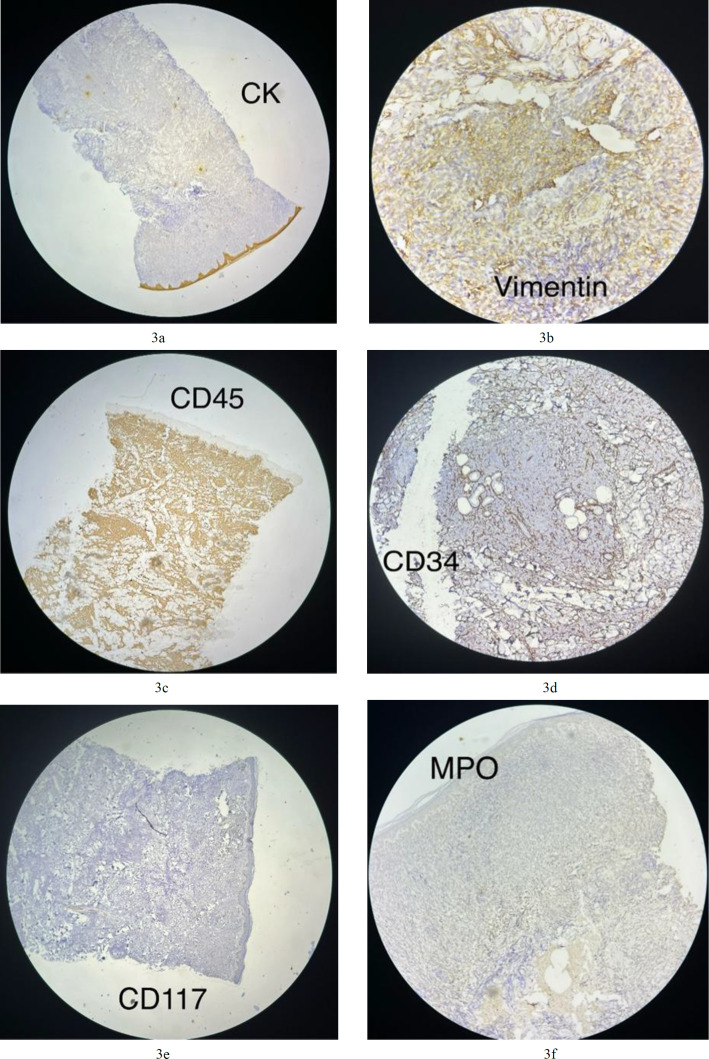
Neoplastic cells negative for CK; **3b.** Some neoplastic cells positive for vimentin (cytoplasmic staining); **3c.** Neoplastic cells are positive for CD45 (cytoplasmic and membranous staining); **3d.** Neoplastic cells are negative for CD34. Endothelial cells are positive for CD34 (membranous staining) as an internal control; **3e.** Neoplastic cells are negative for CD117; Behnoosh.mohamadi.mui@gmail.com Neoplastic cells are negative for MPO

**Fig.3g:**
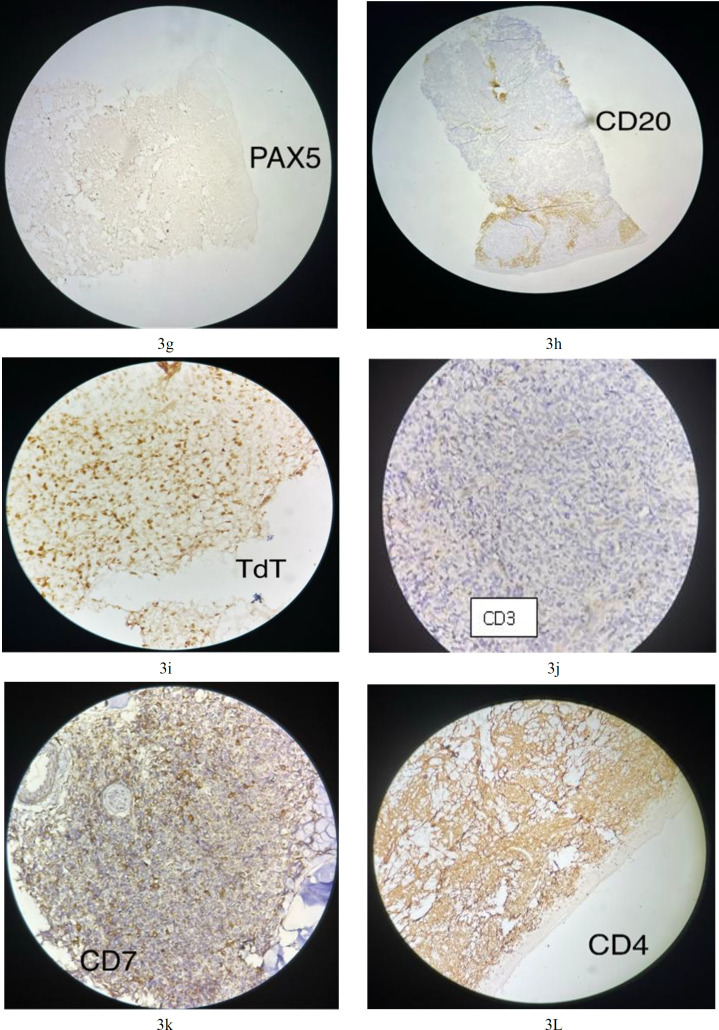
Neoplastic cells are negative for PAX5; **3h.** Neoplastic cells are negative for CD20. Some background cells are positive (cytoplasmic staining); **3i.** Neoplastic cells are positive for TdT (nuclear staining)**;** **3j.** Neoplastic cells are negative for CD3; **3k.** Neoplastic cells are negative for CD7; **3L.** Neoplastic cells are positive for CD4 (cytoplasmic staining)

**Fig. 3m:**
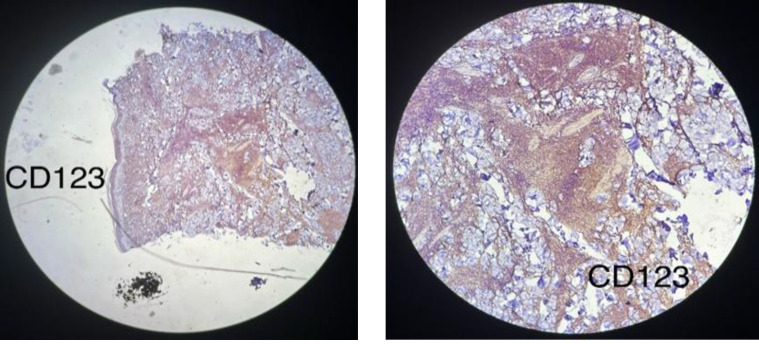
Neoplastic cells are positive for CD123 x4 and x10 magnification (membranous staining)

**Fig.3n:**
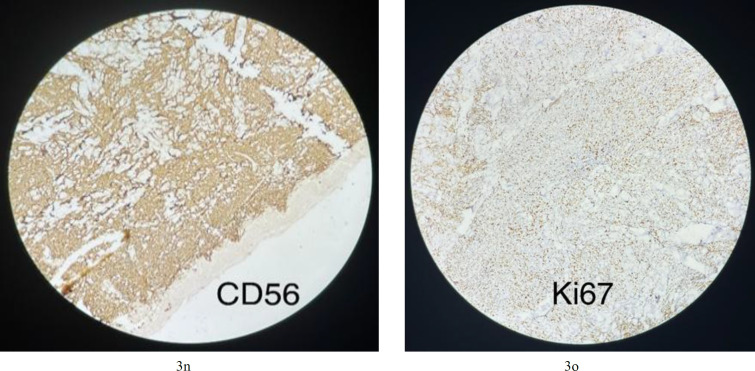
Neoplastic cells are positive for CD56 (membranous staining); **3o. **Neoplastic cells are positive for Ki67 (nuclear staining staining about 50% of cells);

**Fig. 4a:**
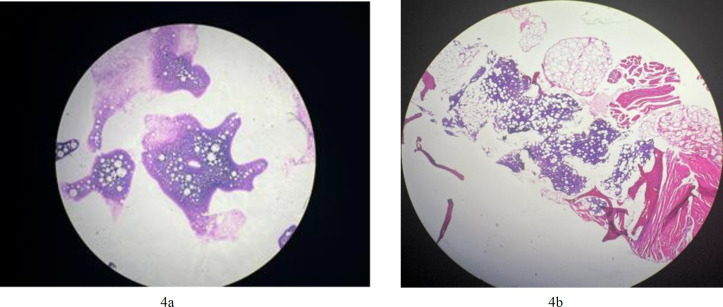
Touch prep smears of bone marrow show some blasts; **4b.** bone marrow biopsy shows variable cellularity with an average of about 50% which was replaced by a monotonous population of mononuclear cells

**Fig.4c:**
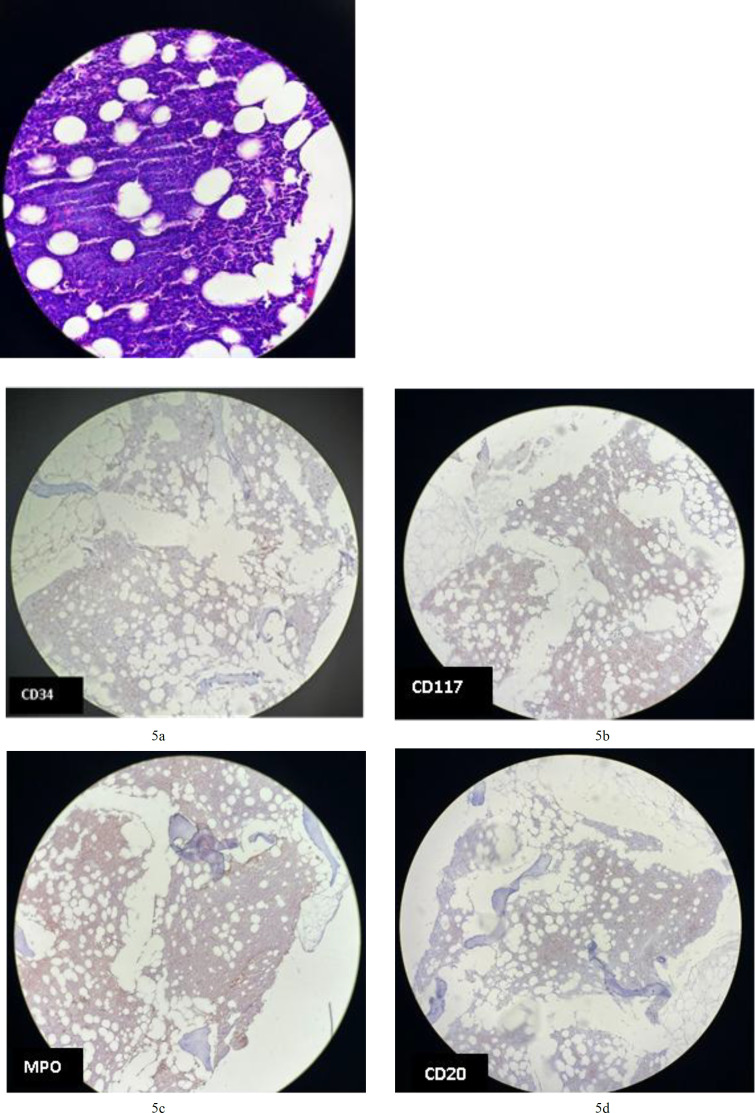
bne marrow biopsy shows variable cellularity with an average of about 50% which was replaced by a monotonous population of mononuclear cells (H&E x40)

**Fig.5a:**
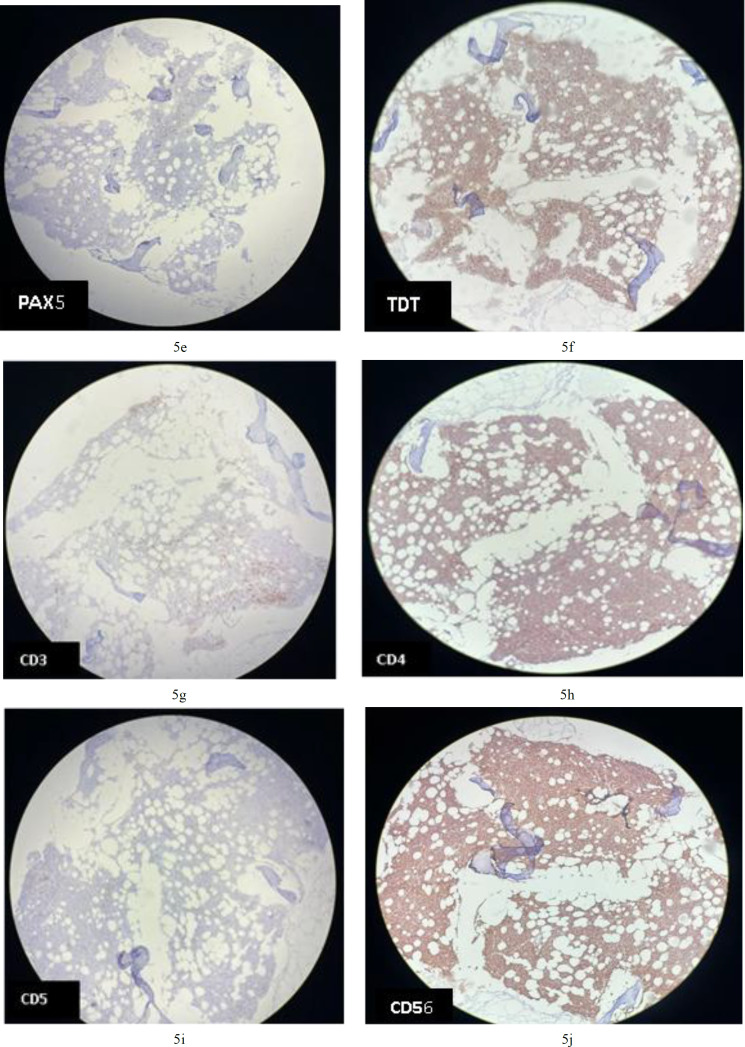
Tumoral cells are negative for CD34 in BM; **5b.** Tumoral cells are negative for CD117 in BM; **5c.** Tumoral cells are negative for MPO in BM; **5d.** Tumoral cells are negative for CD20 in BM; **5e.** Tumoral cells are negative for PAX5 in BM; **5f.** cells are positive for TdT (nuclear staining staining) in BM; **5g.** Tumoral cells are negative for CD3 in BM; **5h.** Tumoral cells are positive for CD4 (cytoplasmic staining) in BM; 5i**. **Tumoral cells are negative for CD5 in BM; **5j.** Tumoral cells are positive for CD 56 (membranous staining) in BM;

**Table 1:** Comparing survival rates over the years is common practice

Case number	Gender	Age	Extracutaneous disease	Survival	therapy	Reference	year
1	male	68	Bone marrow	1 year	chemotherapy	(11)	2022
2	male	54	CNS	3 years	chemotherapy	(12)	2021
3	male	81	Lung	9 months	chemotherapy	(13)	2023
4	male	27	CNS	Alive with no clinical relapse	bone marrow transplantation	(14)	2022
5	female	47	Breast	Alive with no clinical relapse	bone marrow transplantation	(15)	2021
6	male	59	Bone marrow	Alive with no clinical relapse	chemotherapy	(16)	2021
7	male	70	Bone marrow	Response to therapy	chemotherapy	(17)	2019
8	male	70	None	Alive with no clinical relapse	bone marrow transplantation	(18)	2021
9	male	65	Bone marrow	1 year	chemotherapy	(19)	2012
10	male	76	Bone marrow	9 months	chemotherapy	(20)	2014
11	male	49	Bone marrow	17 months	chemotherapy	(21)	2023
12	female	48	-	Alive with no clinical relapse	bone marrow transplantation	(22)	2020
13	male	55	-	1 month	chemotherapy	(22)	2020
14	female	61	-	Alive with no clinical relapse	chemotherapy	(23)	2023
15	male	71	Bone marrow	Few months	chemotherapy	(24)	2020

An initial panel of immunostains showed that the tumor cells were positive for CD45 (cytoplasmic and membranous; Fig 3c) and exhibited scattered positivity for vimentin (cytoplasmic; Fig 3b). They were negative for CD3 (Fig 3j), CD20 (Fig 3h), myeloperoxidase (Fig 3f), PAX5 (Fig 3g), and CK (Fig 3a). Further studies revealed that the cells were positive for CD4 (cytoplasmic; Fig 3l), TdT (nuclear; Fig 3i), CD56 (membranous; Fig 3n), and CD123 (membranous; Fig 3m), and negative for CD34 (Fig 3d), CD117 (Fig 3e), CD7 (Fig 3k), CD10, and CD138.

The Ki-67 proliferative index was approximately 50% (reference value <20%) (nuclear positivity; Fig 3o), which is higher than normal. Based on the morphological and immunohistochemical findings, a diagnosis of BPDCN was made. A staging bone marrow biopsy was performed before treatment initiation, and the bone marrow showed no involvement by tumor cells.

Our patient began receiving an intensive systemic chemotherapy regimen used for acute lymphoblastic leukemia (ALL). The regimen was a modified ALL therapy that included cyclophosphamide, Adriamycin, vincristine, and prednisolone (the CHOP regimen). After the fourth course of chemotherapy, the patient’s skin lesions healed and left scarring, indicating a positive response.

Five months after the ninth course of chemotherapy, she was hospitalized with B symptoms (fever, night sweats, and weight loss) and pancytopenia. Laboratory results revealed a hemoglobin level of 9.3 × 10^10 g/L (normal range, 11.0–15.0 × 10^10 g/L), white blood cell count of 2.3 × 10^9/L (normal range, 4–10 × 10^9/L), and platelet count of 17 × 10^10/L (normal range, 15.0–45.0 × 10^10/L).

During physical examination, she had a temperature of 38°C and no signs of enlarged lymph nodes. A bone marrow biopsy was performed (the patient did not have any bone marrow aspiration smears but had touch prep smears showing some blasts [Fig 4a]). The biopsy showed variable cellularity, averaging about 50%, replaced by a monotonous population of mononuclear cells. Other hematopoietic series and megakaryocytes were decreased. As seen in (Fig 4b), the abnormal bone marrow cells had a relatively moderate nuclear-to-cytoplasmic ratio, oval nuclear contours, numerous nuclei with incisura, and coarse chromatin (Fig 4c).

Immunohistochemical analysis revealed that the CD123-positive cells co-expressed CD4 (cytoplasmic staining; Fig 5h) and CD56 (membranous staining; Fig 5j). They were partially positive for terminal deoxynucleotidyl transferase and negative for myeloperoxidase (MPO; Fig 5c), CD34 (Fig 5a), CD117 (Fig 5b), CD20 (Fig 5d), and CD3 (Fig 5g). Diagnosis of BPDCN was indicated by positivity for CD4 and CD56, along with other markers more specific to plasmacytoid dendritic cells, and negativity for lymphoid, NK, and myeloid lineage-associated antigens.

During hospitalization, the patient experienced loss of consciousness and respiratory distress and was admitted to the ICU, where she subsequently died.

## Discussion

Blastic plasmacytoid dendritic cell neoplasms usually affect patients between 60 and 70 years of age, although they can develop at any age, including childhood. This condition is more prevalent in males, with a ratio of 3:1 compared to females (3).

BPDCN is characterized by numerous cutaneous lesions at diagnosis, along with extracutaneous involvement of the bone marrow, peripheral blood, and lymph nodes. Most patients present with one or multiple asymptomatic skin lesions that may appear as nodules, plaques, or bruise-like marks, ranging in size from a few millimeters to as large as 10 cm. Upon diagnosis, most BPDCN patients already have extracutaneous disease, often affecting regional lymph nodes. As the disease progresses, peripheral blood and bone marrow involvement may also develop (5).

Peripheral cytopenia is frequently mild to moderate at diagnosis, and patients rarely exhibit systemic symptoms. As BPDCN advances, fulminant leukemia can develop regardless of the presence of cutaneous lesions. Coincident myelodysplasia is identified in 10–20% of BPDCN patients, potentially leading to acute myelomonocytic leukemia or AML (6).

BPDCN without cutaneous lesions typically occurs in younger patients who present with marked leukocytosis, anemia, and thrombocytopenia, resulting in a notably reduced median survival. Involvement of peripheral blood and bone marrow tends to appear as the disease progresses. Fulminant leukemia at initial presentation is rare. Although lymph node (LN) involvement is frequent, splenomegaly and mucosal involvement are relatively uncommon. Systemic B symptoms are not often seen at diagnosis (7).

In BPDCN, lymph nodes may exhibit diffuse involvement and complete effacement of their architecture. Histologically, a monotonous population of small to medium cells with irregular nuclear contours, finely dispersed chromatin, 1–3 small nucleoli, and scant to moderate cytoplasm is observed. Extensive bone marrow involvement may lead to neoplastic cells in the peripheral blood, resembling circulating lymphoid or myeloid blasts. In bone marrow aspirates, these neoplastic cells may show “pearl necklace–like” submembranous cytoplasmic vacuoles and elongated, agranular cytoplasm. Dysplastic changes can also occur in megakaryocytes, granulocytes, and erythroid precursors in both the bone marrow and peripheral blood (7).

A definitive BPDCN diagnosis relies on clinical features, morphology, immunophenotyping, and cytogenetic/molecular data. Immunophenotyping is particularly crucial due to similarities with other hematopoietic neoplasms; consequently, a thorough analysis is necessary. Before establishing a diagnosis of BPDCN, it is important to exclude myeloid sarcoma/AML, T-cell lymphoblastic leukemia/lymphoma (T-ALL/LBL), NK-cell lymphoma/leukemia, and certain mature T-cell lymphomas/leukemias (5).

To confirm BPDCN, its specific immunophenotype must be demonstrated by immunohistochemistry or flow cytometry, in line with WHO 2022 criteria:

- Expression of CD123 and one other pDC (plasmacytoid dendritic cell) marker in addition to CD4 and/or CD56, OR
- Expression of any three pDC markers and absence of all expected negative markers.

Expected positive pDC markers include CD123, TCF4, TCL1, CD303, CD304, CD4, and CD56, while expected negative markers include CD3, CD14, CD19, CD34, lysozyme, and myeloperoxidase (4).

BPDCN is associated with multiple, recurrent genetic abnormalities, although no single cytogenetic alteration is definitively diagnostic (8). Evidence shows a recurring pattern of deletions involving tumor suppressor genes such as RB1, CDKN1B, CDKN2A, and TP53. The TET2 gene (Ten–Eleven translocation–2) located on 4q24 is often mutated in BPDCN, as well as in myelodysplastic syndromes, chronic myelomonocytic leukemia, and AML, suggesting that BPDCN is a myeloid neoplasm (5).

When considering BPDCN, it is essential to distinguish it from other conditions with mature plasmacytoid dendritic cells, such as Kikuchi-Fujimoto disease and chronic myelomonocytic leukemia. In those disorders, mature plasmacytoid dendritic cells lacking CD56 do not indicate BPDCN involvement. Other differential diagnoses include CD4+/CD56+ diseases that manifest in the skin. However, immunophenotyping typically allows for differentiation. For example, nasal-type extranodal NK/T-cell lymphoma—unlike BPDCN—demonstrates Epstein-Barr virus–encoded small RNAs via in situ hybridization. Both diseases can express T-cell antigens and CD56, but extranodal NK/T-cell lymphoma typically shows angiocentric, angio-destructive growth and cytoplasmic CD3 (ε chain) expression, along with cytotoxic molecules. BPDCN does not exhibit these characteristics. Likewise, cutaneous monocytic leukemias express lysozyme, absent in BPDCN (5,8).

Even patients who achieve complete remission generally have a poor prognosis, with median survival ranging from 12 to 27 months in various studies. However, some series involving pediatric patients or those with cutaneous-only disease report higher overall survival rates. Patients treated with ALL/lymphoma-type regimens often have better outcomes than those receiving AML-type treatment. Standard therapy typically involves multiagent chemotherapy, such as CHOP (cyclophosphamide, doxorubicin, vincristine, prednisone) or hyper-CVAD (with alternating cycles of cyclophosphamide, vincristine, doxorubicin, and methotrexate/cytarabine). Although initial chemotherapy often results in high complete response rates (47–86%), relapses are common, and the relapsed disease is usually resistant to prior agents. Recent clinical evidence suggests that some elderly patients may achieve durable remission with high-dose chemotherapy followed by an allogeneic stem cell transplant from a matched related or unrelated donor (9).

In contrast to adults, pediatric BPDCN generally presents less aggressively. High-risk acute lymphoblastic leukemia protocols have proven effective in treating BPDCN in children. Stem cell transplantation is reserved for pediatric patients whose disease fails to enter complete remission or who experience relapse (10).

Comparing survival rates over the years is common practice, given the disease’s overall poor prognosis. This comparison aims to clarify life expectancy among patients affected by BPDCN (Table 1).

## Conclusion

Blastic plasmacytoid dendritic cell neoplasm is a rare acute myeloid leukemia–related precursor neoplasm, as classified by the World Health Organization in 2022. Definitive diagnosis relies on clinical features, morphology, immunophenotyping, and cytogenetic/molecular data. Due to its similarities with other hematopoietic neoplasms, a thorough analysis is necessary to exclude myeloid sarcoma/AML, T-cell lymphoblastic leukemia/lymphoma (T-ALL/LBL), NK-cell lymphoma/leukemia, and certain mature T-cell lymphomas/leukemias.
